# Surface Plasmon Resonance for Protease Detection by Integration of Homogeneous Reaction

**DOI:** 10.3390/bios11100362

**Published:** 2021-09-29

**Authors:** Ning Xia, Gang Liu, Xinyao Yi

**Affiliations:** 1 College of Chemistry and Chemical Engineering, Anyang Normal University, Anyang 455000, China; ningxia@aynu.edu.cn (N.X.); liugang08215@163.com (G.L.); 2 College of Chemistry and Chemical Engineering, Henan University of Technology, Zhengzhou 450011, China; 3 College of Chemistry and Chemical Engineering, Central South University, Changsha 410083, China

**Keywords:** surface plasmon resonance, protease, caspase, avidin-biotin interaction

## Abstract

The heterogeneous assays of proteases usually require the immobilization of peptide substrates on the solid surface for enzymatic hydrolysis reactions. However, immobilization of peptides on the solid surface may cause a steric hindrance to prevent the interaction between the substrate and the active center of protease, thus limiting the enzymatic cleavage of the peptide. In this work, we reported a heterogeneous surface plasmon resonance (SPR) method for protease detection by integration of homogeneous reaction. The sensitivity was enhanced by the signal amplification of streptavidin (SA)-conjugated immunoglobulin G (SA-IgG). Caspase-3 (Cas-3) was determined as the model. A peptide labeled with two biotin tags at the N- and C-terminals (bio-GDEVDGK-bio) was used as the substrate. In the absence of Cas-3, the substrate peptide was captured by neutravidin (NA)-covered SPR chip to facilitate the attachment of SA-IgG by the avidin-biotin interaction. However, once the peptide substrate was digested by Cas-3 in the aqueous phase, the products of bio-GDEVD and GK-bio would compete with the substrate to bond NA on the chip surface, thus limiting the attachment of SA-IgG. The method integrated the advantages of both heterogeneous and homogeneous assays and has been used to determine Cas-3 inhibitor and evaluate cell apoptosis with satisfactory results.

## 1. Introduction

Proteases play an important role in a wide variety of biological processes, including protein digestion, wound healing, apoptosis, fertilization, growth differentiation, and immune system activation [1]. In the human body, at least 1.7% of human genes are encoded by proteases. The activities of proteases are closely related to many diseases, such as cancer, cardiovascular disease, Alzheimer’s disease, human immunodeficiency virus (HIV), thrombosis, and diabetes [2]. Thus, extensive efforts have been made to screen protease inhibitors as potential drugs. This provides a powerful motivation for the development of sensitive, selective, and robust methods to detect protease and discover potential inhibitors.

Until now, many homogeneous and heterogeneous biosensors have been reported for the detection of proteases and screening of their inhibitors [2,3]. In homogeneous analysis, the substrate and protease sample are present in the aqueous phase. For instance, in the fluorescence resonance energy transfer (FRET) assay, the commonly used method for protease activity detection, the peptides labeled with two different fluorophores at two ends are digested by protease in the aqueous phase [4]. The activity of protease can be measured by monitoring the change of fluorescence signal after the cleavage of the peptide. On the contrary, the peptide substrate is anchored on a solid surface in the heterogeneous assay, and the enzymatic reaction happens at the solid-liquid interface [5,6]. Both the homogeneous and heterogeneous methods have their own advantages and disadvantages. Usually, homogeneous biosensors have the advantages of easy operation, rapid response, excellent sensitivity, and high throughput, but they show poor anti-interference ability and require large sample volumes and complex sample handling procedures. Conversely, heterogeneous assays exhibit the advantages of less sample consumption, ultra-high sensitivity and selectivity, and low instrument investment. Overall, the heterogeneous biosensors provide tremendous advantages over conventional homogeneous assays since numerous peptide substrates are immobilized at a discrete location on the solid interface [2]. However, immobilization of peptides on the solid surface will cause a steric hindrance to prevent the interaction between the substrate and the active center of protease [7], thus limiting the enzymatic cleavage of the peptide. Although the steric hindrance can be reduced by the use of nanomaterials-modified interface and the well-design of peptide substrate [8,9,10], the surface chemistry and coverage of peptide on the solid surface demands laborious optimization. Therefore, it is of importance to integrate the advantages of both heterogeneous and homogeneous assays for the design of general protease biosensors.

**Scheme 1:**
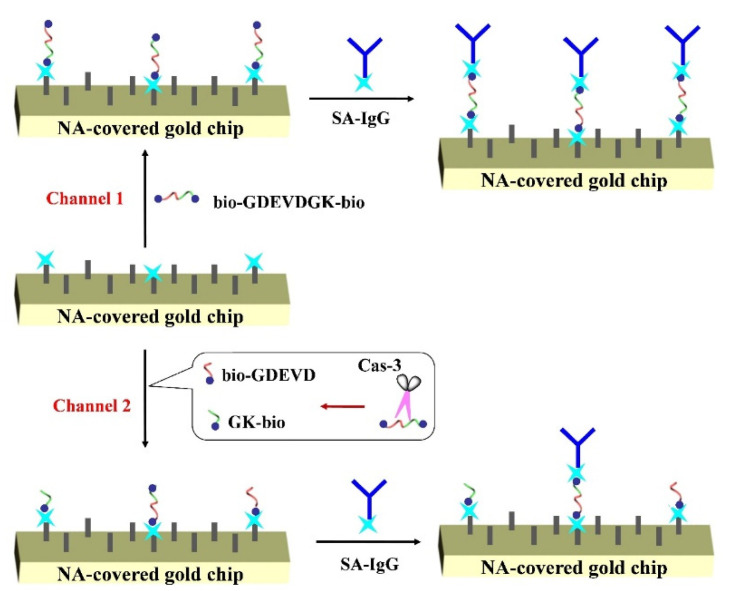
Schematic representation of SPR method for the detection of Cas-3 using NA-covered gold chip. The signal was amplified by SA-IgG conjugates.

Surface plasmon resonance (SPR) is a simple, label-free technology to monitor the protein-protein interactions by measuring the refractive index change at the sensor surface [11,12,13,14]. The technology can be used to monitor the cleavage of protein or peptide fixed on the chip surface, providing a label-free detection method for protease analysis due to the advantages of fast response, real-time detection, high signal-to-noise, and good compatibility with the microfluidic system. For example, Steinrücke and co-workers suggested that cleavage of the helical protein with 78 amino acids by protease caused a detectable SPR signal [15]. However, the cleavage of low molecular weight peptides leads to a small, undetectable change in the refractive index [16,17,18]. Thus, it usually requires a signal amplification strategy to detect protease by labeling the peptide substrate with nanomaterials or specific groups [19,20,21,22,23,24]. Biotin is usually used to label peptide substrate for the design of heterogeneous biosensors. It can interact with avidin or its analogs of neutravidin (NA) and streptavidin (SA) with a binding coefficient as high as ~10^15^ M^−1^. Such an interaction allows for the immobilization and recognition of peptide substrate at the solid-liquid interface [25,26,27,28]. By integrating the advantages of homogeneous assays, herein, we proposed a novel SPR method for protease detection by the signal amplification of SA-conjugated immunoglobulin G (SA-IgG). Caspases, a family of cysteine proteases, play an important role in apoptosis. To demonstrate the feasibility of the method, caspase-3 (Cas-3) that can specifically recognize and cleave the C-terminal of the peptide with the DEVD sequence was determined as the model. A peptide labeled with two biotin tags at the N- and C-terminals (bio-GDEVDGK-bio) was used as the substrate (Scheme 1). In the absence of Cas-3, the peptide substrate can be captured by the NA-covered chip through the avidin-biotin interaction (Channel 1). The biotin group at the other end of the peptide allows for the capture of SA-IgG, thus resulting in a strong SPR signal. When the peptide substrate was digested by Cas-3 in the aqueous phase, the biotinylated products (bio-GDEVD and GK-bio) would compete with the substrate to bond NA on the chip surface (Channel 2). This prevents the attachment of bio-GDEVDGK-bio and the follow-up capture of SA-IgG by the avidin-biotin interaction. However, when the activity of Cas-3 was suppressed by inhibitor, more bio-GDEVDGK-bio substrates would be anchored on the chip surface, which facilitating the capture of SA-IgG. The method was used to evaluate the inhibition efficiency of the inhibitor and monitor the activity of Cas-3 in cell lysates.

## 2. Materials and Methods

### 2.1. Chemicals and Materials

NA protein was purchased from Thermo Fisher Scientific (Shanghai, China). Cas-3 was obtained from New England BioLabs (Ipswich, MA, USA). Thrombin, beta-secretase, prostate-specific antigen (PSA), and bovine serum protein (BSA) were acquired from Sigma-Aldrich (Shanghai, China). SA-IgG and glutathione (GSH) were purchased from Sangon Biotech (Shanghai, China). Peptides were provided by China Peptide Co., Ltd. (Shanghai, China). Other reagents were ordered from Aladdin Reagent Co., Ltd. (Shanghai, China). All aqueous solutions were prepared daily with ultrapure water collected from a Milli-Q purification system.

### 2.2. Preparation of SPR Chips

The gold chips were annealed in a hydrogen flame to eliminate the surface contaminant. Then, the cleaned gold chips were incubated with 1 μM NA protein in carbonate buffer (pH 10) for 2 h. NA was capped on the gold surface through the hydrophobic and Au-S interactions [29]. The unbound NA proteins were removed by rinsing the chip with the carbonate buffer. The unreacted gold surface were blocked by incubation of the chip with 10 μM BSA and 100 μM GSH. Finally, the NA-covered chips were thoroughly washed with the carbonate buffer.

### 2.3. Procedure for Cas-3 Detection

The peptide bio-GDEVDGK-bio was digested by Cas-3 in the HEPES buffer with the optimized reaction conditions. The reaction mixture was delivered to the flow cell by a syringe pump. When the baseline is stable, SA-IgG in phosphate buffer (10 mM, pH 7.4) was injected into the SPR channel by the pump. The signal was collected by measuring the change of SPR dip shift on a BI-SPR 3000 system (Biosensing Instrument Inc., Tempe, AZ, USA).

### 2.4. Inhibitor Detection and Cell Lysate Analysis

For the detection of Cas-3 inhibitor, DEVD-FMK at different concentrations was mixed with a given concentration of Cas-3 for 10 min. Then, the resultant solution was incubated with the peptide substrate. For real sample assays, HeLa cells were cultured and the cell lysates were extracted with our reported procedures [30,31]. Then, the peptide substrate was incubated with the diluted cell lysates to react for 2 h. Finally, the reaction mixture was delivered to the flow cell, followed by injection of SA-IgG to the channel after the baseline stable was attained.

## 3. Results and Discussion

### 3.1. Feasibility of the Strategy

**Figure 1:**
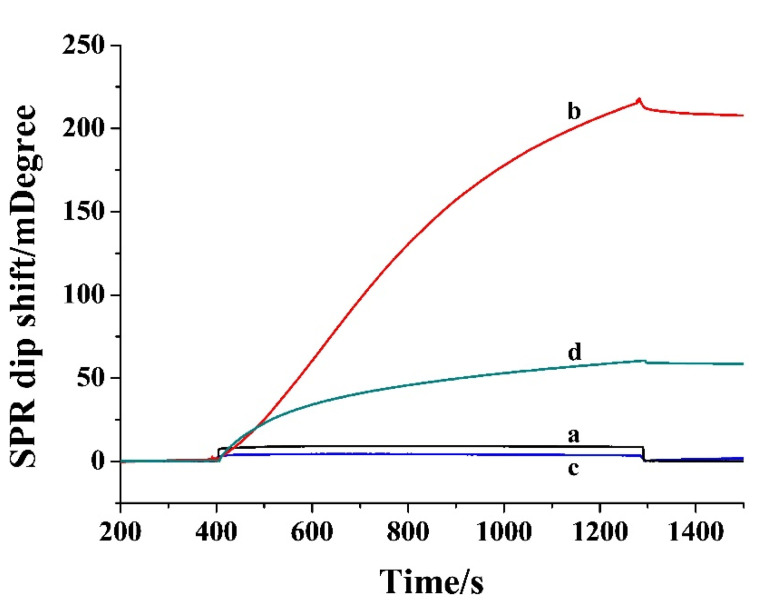
SPR sensorgrams when injecting 0.1 mg/mL SA-IgG to the fluidic channel wherein the NA-covered chip had been treated by blank buffer (curve a) and 20 nM bio-GDEVDGK-bio (curve b). Curves c and d were acquired when injecting 0.1 mg/mL IgG and 0.05 mg/mL SA onto the NA-covered chip treated by bio-GDEVDGK-bio.

Based on the avidin-biotin interactions, NA or SA-modified magnetic beads, chromatography columns and solid surfaces have been widely used for the immobilization and separation of biotinylated biomolecules. In this work, an NA-covered gold chip was used to capture the biotinylated peptide. Figure 1 depicts the SPR responses when injecting SA-IgG, SA, and IgG to the sensor channel. A negligible change in the dip shift was observed after injecting SA-IgG conjugate to the NA-covered chip (curve a), demonstrating that SA-IgG showed no interaction with the sensor chip. Interestingly, the SPR dip shift reached 207 mD when injecting the conjugate to the chip treated by bio-GDEVDGK-bio (curve b). No significant change was observed when injecting IgG onto the bio-GDEVDGK-bio treated channel (curve c) and a smaller SPR dip shift (59 mD) was attained when injecting SA onto the channel (curve d). Thus, the change in curve b should be attributed to the avidin-biotin interaction and the signal was greatly amplified by IgG due to its large molecular weight (~150000 Da). We also found that the signal was intensified with the increase in bio-GDEVDGK-bioconcentration from 0.01 to 20 nM and began to level off beyond 5 nM. To attain higher sensitivity and a wider linear range, 5 nM bio-GDEVDGK-bio was used as the substrate for the assays of Cas-3.

### 3.2. Detection of Cas-3 and Its Inhibitor

**Figure 2:**
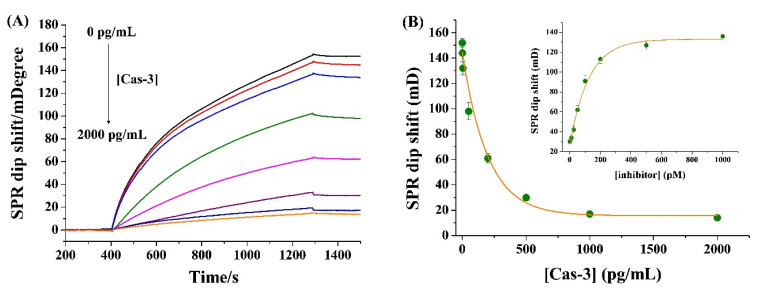
(**A**) SPR sensorgrams when injecting 0.02 mg/mL SA-IgG onto the NA-covered chips treated by the mixture of 5 nM bio-GDEVDGK-bio and a given concentration of Cas-3 (from top to bottom: 0, 0.5, 5, 50, 200, 500, 1000, and 2000 pg/mL). (**B**) Dependence of SPR signal on the concentration of Cas-3. The inset shows the effect of inhibitor concentration on the SPR signal wherein the concentration of Cas-3 was 500 pg/mL.

The analytical performances were first investigated by determining different concentrations of Cas-3. In Figure 2A, the SPR signal decreased gradually with the increase in Cas-3 concentration in the range of 0~2000 pg/mL. The plateau beyond 1000 pg/mL is indicative of the completion of the enzymatic hydrolysis (Figure 2B). The signal did not decrease to the background value, indicating that not all the substrate peptides were cleaved by Cas-3 even at a higher concentration with a very long reaction time. The detection limit was estimated to be 0.5 pg/mL by measuring the sensor response to a dilution series and determining the smallest target concentration at which the signal was clearly distinguishable from the response to the blank solution. The value is lower than that attained by the homogeneous methods, such as fluorescence (128 pg/mL) [32], colorimetric assay (5 ng/mL) [33], differential pulse voltammetry (27.4 ng/mL) [34], and mass spectrometry (3.02 ng/mL) [35]. The value is comparable to or even lower than that achieved by heterogeneous methods based on the signal amplification of enzymes and nanomaterials (Table 1). The high sensitivity of the method should be attributed to the “immobilization-free” hydrolysis reaction and the large molecular weight of SA-IgG.

As a proof-of-concept experimental for evaluation of Cas-3 activity, the inhibitor DEVD-FMK at different concentrations was incubated with 500 pg/mL Cas-3. The inset in Figure 2B shows the dependence of SPR dip shift on the concentration of inhibitor. The increase in inhibitor concentration induced the enhancement in the SPR signal, indicating that DEVD-FMK is an effective Cas-3 inhibitor. The half-maximal inhibitory concentration (IC_50_) was estimated to be 98 nM, which is in agreement with that measured by other methods [30,36]. Thus, the method has bright prospects for the screening of protease inhibitors.

### 3.3. Selectivity

**Figure 3:**
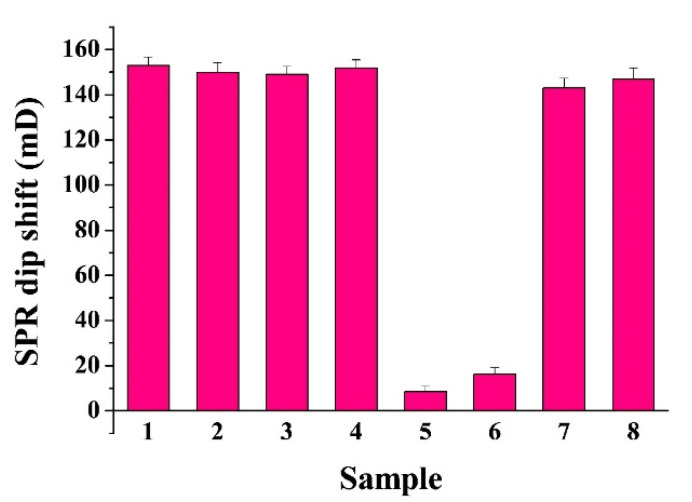
Selectivity of the method: bar 1, thrombin; bar 2, beta-secretase; bar 3, PSA, bar 4, Cas-3; bar 5, avidin; bar 6, bio-GLRRASLG; bar 7, the mixture of avidin and biotin pretreated by SA-modified magnetic beads; bar 8, bio-GLRRASLG pretreated by SA-modified magnetic beads. The concentrations of thrombin, beta-secretase, PSA, Cas-3, avidin, biotin, and bio-GLRRASLG were 5 ng/mL, 5 ng/mL, 5 ng/mL, 500 pg/mL, 200 ng/mL, 12.5 nM, and 5 nM, respectively.

To evaluate the specificity of the method, the method was first challenged by determining other proteases (e.g., thrombin, beta-secretase, and PSA) to replace Cas-3. As shown in Figure 3, the tested proteases did not induce a significant decrease in the SPR dip shift, suggesting that the method shows high selectivity toward Cas-3 (*cf.* curves 1~4). However, trace biotin or other molecules in real samples may interact with biotin, thereby limiting the practical application of the technique. For this consideration, the interferences from avidin and biotinylated biomolecule such as bio-GLRRASLG were examined. As envisaged, both avidin and bio-GLRRASLG caused a significant decrease in the SPR signal (curves 5~6). The result is understandable as avidin can bind to the peptide substrate (bio-GDEVDGK-bio) and the biotinylated peptide can compete with the substrate to bind NA on the chip surface, thus preventing the attachment of bio-GDEVDGK-bio on the chip. To resolve this problem, a certain amount of biotin was added to the sample in advance to eliminate the interference of avidin. The free biotin or biotinylated peptide was then removed by the commercial SA-modified magnetic beads. As a result, the interferences from avidin and biotinylated peptide have been well eliminated (curves 7~8).

### 3.4. Evaluation of Cell Apoptosis

**Figure 4:**
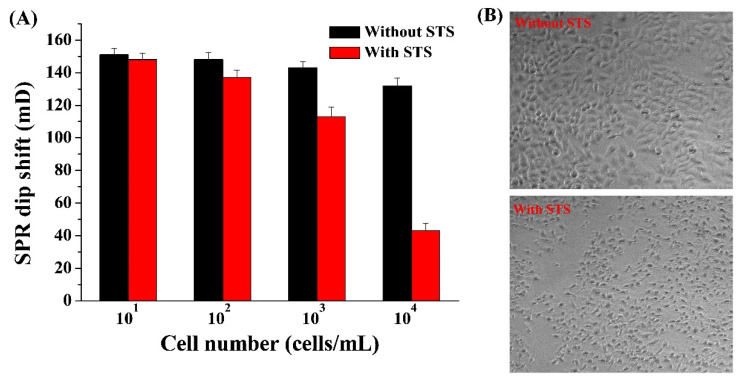
(**A**) Dependence of SPR signal on the concentration of normal and STS-treated HeLa cells. (**B**) Confocal images of normal and STS-treated HeLa cells.

Apoptosis is a highly regulated physiological process, which is of great significance in the life cycle of organisms. However, the imbalance of apoptosis may directly lead to the occurrence of many diseases. Therefore, the death caused by apoptosis has attracted extensive attention from experts in pathology, pharmacology, and toxicology. Among various types of caspases, Cas-3 is the central molecule to mediate the apoptotic pathway inside and outside cells. Therefore, Cas-3 has been regarded as the biomarker and therapeutic target for the diagnosis and treatment of apoptosis-related diseases. To verify the feasibility of this method for monitoring cell apoptosis, HeLa cells were used as the models. As shown in Figure 4A, when the peptide substrates were incubated with the cell lysates extracted from normal HeLa cells, the SPR signals were high and no significant changes were observed with the increase in cell number. However, when the cells were treated by STS (a common apoptosis inducer), the SPR signals decreased gradually with the increase in cell number. This indicated that the apoptosis was triggered by STS and the activity of Cas-3 was activated during apoptosis. STS-induced apoptosis was also confirmed by characterizing the cell morphology with a microscope (Figure 4B). The result is consistent with that obtained by other methods, indicating that the method can be used for the evaluation of apoptosis by monitoring the Cas-3 activity.

## 4. Conclusions

In summary, we reported a heterogeneous SPR method for protease detection by integration of homogeneous enzymatic hydrolysis reaction. The signal was amplified by SA-IgG because of its large molecular weight. The method was used to determine Cas-3 activity and evaluate cell apoptosis with satisfactory results. The method exhibited high sensitivity and obviated the use of enzymes or nanomaterial for signal amplification. The “immobilization-free” strategy for the enzymatic reaction should be valuable for the design of novel heterogeneous biosensors to eliminate the effect of steric hindrance.

## Figures and Tables

**Table 1:** An overview of heterogeneous methods for Cas-3 detection based on the signal amplification of enzymes and nanomaterials.

Method	Signal Label	Linear Range	Detection Limit	Ref.
EIS	Biotin-FNP network	1–125 pg/mL	1 pg/mL	[30]
EIS	SA-peptide network	0–50 pg/mL	0.2 pg/mL	[36]
DPV	Methylene blue/GO	0.1–100 pg/mL	0.06 pg/mL	[37]
DPV	Calixarene-rGO	10–100 pg/mL	0.0167 pg/mL	[38]
DPV	AuNPs-MCM/MB-HRP	10 fM–10 nM	5 fM	[39]
LSV	CB(8)-capped AgNPs	1–10 ng/mL	24.62 pg/mL	[40]
SWV	MB/HRP	0.1–1 nM	56 pM	[41]
ECL	Ru@SiO_2_	0.2–200 pg/mL	0.07 pg/mL	[42]
ECL	HRP-SA-MB	0.5–100 fM	0.5 fM	[43]

Abbreviations: DPV, differential pulse voltammetry; LSV, linear sweep voltammetry; SWV, square wave voltammetry; EIS, electrochemical impedance spectroscopy; ECL, electrochemiluminescence; GO, graphene oxide; rGO, reduced graphene oxide; AuNPs-MCM, gold nanoparticle-coated silica-based mesoporous materials; MB, magnetic bead; HRP, horseradish peroxidase; AgNPs, silver nanoparticles; FNP, self-assembled biotin-phenylalanine nanoparticle.

## Data Availability

Not applicable.
